# Adjuvant chemotherapy is an additional option for locally advanced gastric cancer after radical gastrectomy with D2 lymphadenectomy: a retrospective control study

**DOI:** 10.1186/s12885-021-08717-4

**Published:** 2021-08-30

**Authors:** Lei Chen, Chenghai Zhang, Zhendan Yao, Ming Cui, Jiadi Xing, Hong Yang, Nan Zhang, Maoxing Liu, Kai Xu, Fei Tan, Yuzhe Li, Beihai Jiang, Xiangqian Su

**Affiliations:** Key Laboratory of Carcinogenesis and Translational Research (Ministry of Education), Department of Gastrointestinal Surgery IV, Peking University Cancer Hospital & Institute, 52 Fucheng Road, Haidian District, Beijing, 100142 China

**Keywords:** Adjuvant chemotherapy, Gastric cancer, Survival

## Abstract

**Background:**

This study compared the long-term efficacy of different durations of adjuvant chemotherapy for patients with gastric cancer after radical gastrectomy with D2 lymphadenectomy.

**Methods:**

We retrospectively identified 428 patients with stage II–III gastric cancer who underwent D2 gastrectomy between 2009 and 2016. Patients were divided into four groups according to the duration of adjuvant chemotherapy, including 0 week (no adjuvant, group A), 20 to 24 weeks (completed 7–8 cycles every 3 weeks or 10–12 cycles every 2 weeks, group B), and 12 to18 weeks (completed 4–6 cycles every 3 weeks or 6–9 cycles every 2 weeks, group C), and less than 12 weeks (received up to 3 cycles every 3 weeks or 5 cycles every 2 weeks, group D). The chemotherapy regimens included XELOX, SOX, and FOLFOX. 5-year overall survival (OS) and disease-free survival (DFS) were analyzed.

**Results:**

The 5-year OS rates for groups A, B, C, and D were 52.3, 73.7, 72.0, and 53.3%, respectively, and the 5-year DFS rates were 50.0, 68.0, 65.4, and 50.0%, respectively. OS and DFS were higher in group B than in groups A and D. Similarly, patients in group C were more likely to have higher OS and DFS than those in groups A and D. Meanwhile, there were no significant differences in OS and DFS between groups B and C. The multivariate analysis confirmed with high statistical significance the efficacy of complete courses of adjuvant chemotherapy, and, among them, the similar impact of 4–6/6–9 and 7–8/10–12 cycles, resulting in similar HRs vs Group A (0.52 and 0.42, respectively).

**Conclusions:**

To reduce toxicity and maintain efficacy, XELOX or SOX chemotherapy regimens administered for 4–6 cycles every 3 weeks or FOLFOX regimen for 6–9 cycles every 2 weeks might be a favorable option for patients with stage II–III gastric cancer after D2 gastrectomy. Prospective multicenter clinical trials with adequate sample sizes are necessary to verify these findings.

## Background

Gastric cancer (GC) is one of the most common malignancies in humans, ranking fifth in incidence and third in mortality globally. Geographically, Eastern Asia has the highest incidence and mortality rates of GC in the world, and the disease is mainly concentrated in Korea, China, and Japan [1]. In China, although the overall incidence of GC is declining, it remains the second deadliest malignancy after lung cancer and the third leading cause of mortality [2]. Radical gastrectomy with D2 lymphadenectomy remains the foundation of curative therapy. However, unlike the situation in Japan and Korea, more than 80% of patients with GC in China are diagnosed with locally advanced disease, which carries higher risks of postoperative recurrence and metastasis. Therefore, postoperative adjuvant chemotherapy is the main treatment [3].

Since 2001, several large clinical studies have provided high-level evidence of the benefits of adjuvant chemotherapy in GC [4–7]. Currently, there is no global agreement concerning chemotherapy regimens and durations for GC. In Northern Europe, three cycles each of preoperative and postoperative epirubicin, cisplatin, and infused fluorouracil (FU) comprise the accepted regimen, as supported by the MAGIC trial [5]. In Japan, a 6-week cycle of the oral fluoropyrimidine derivative S-1 is repeated for 1 year, in line with the findings of the ACTS-GC study [6]. South Korea, China, and Taiwan favor 6 months of capecitabine and oxaliplatin (XELOX, administered every 3 weeks), in line with the results of the CLASSIC trial [7].

In our center, in accordance with the Chinese Society of Clinical Oncology clinical guidelines, the recommended postoperative adjuvant chemotherapy regimens for patients with stage II–III GC who did not receive preoperative treatment include XELOX, S-1, S-1 and oxaliplatin (SOX, administered every 3 weeks) and FU, oxaliplatin and leucovorin calcium (FOLFOX, administered every 2 weeks) [8]. Although 6 months of XELOX, SOX, or FOLFOX treatment is recommended, the duration of chemotherapy in clinical practice is largely dependent on patients’ compliance and tolerance to adverse events of treatment, such as sensory neurotoxicity, neutropenia, thrombocytopenia, nausea/vomiting, hepatic toxicity, stomatitis, and hand-foot syndrome, with a subset of patients without severe chemotherapy side effects. Moreover, the recommended duration of chemotherapy is expressed as a range of months, as opposed to an exact number. Recently, several studies on the duration of chemotherapy in GC drew inconsistent conclusions [9–13]. Reducing the duration of chemotherapy may increase the risk of recurrence, but some patients are unable to complete a sufficient treatment course. Hence, we conducted a retrospective study of this paradox in the clinical setting to compare the long-term effects of different durations (four groups) of adjuvant chemotherapy on OS for patients with GC after radical gastrectomy with D2 lymphadenectomy. We hope to obtain additional evidence from this study to guide clinical research.

## Methods

Patients treated at Peking University Cancer Hospital & Institute (Beijing, China) from 2009 to 2016 were retrospectively included. All patients had a histologic diagnosis of GC. The clinicopathological features and stage of the patients were determined according to the 8th American Joint Committee on Cancer classification guidelines. Written informed consent to data treatment was obtained from all patients and the study was approved and supervised by the Research Ethics Committee of Peking University Cancer Hospital & Institute. The inclusion criteria were as follows: (1) age, 18–79 years; (2) no obvious surgical contraindications identified in a preoperative multidisciplinary evaluation, such as severe heart or lung disease; (3) prior completion of radical gastrectomy with D2 lymphadenectomy and a postoperative pathological diagnosis of stage II–III gastric cancer; (4) no receipt of adjuvant chemotherapy or receipt of only doublet chemotherapy (XELOX, SOX, or FOLFOX) after surgery; and (5) started adjuvant chemotherapy within 3 months after surgery. The exclusion criteria were as follows: (1) history of malignancy; (2) receipt of adjuvant radiation or neoadjuvant chemotherapy; (3) death within 3 months after surgery, and (4) receipt of monotherapy or triplet chemotherapy regimens.

Six months of XELOX, SOX, or FOLFOX treatment is recommended according to the Chinese Society of Clinical Oncology clinical guidelines [8]. Qu et al. [13] conducted a retrospective analysis of 237 patients with stage IB–IIIC GC showed that six cycles of FU-based adjuvant chemotherapy (18 weeks) are adequate compared to eight cycles. Based on the IDEA trial, 3 months of treatment with CAPOX has been introduced for stage III colon cancer patients in the lower-risk group without sacrificing efficacy [14]. Therefore, we chose 18 weeks and 12 weeks as the grouping nodes and divided the enrolled patients into 4 groups, including 0 week (no adjuvant, group A), 20 to 24 weeks (group B), and 12 to 18 weeks (group C), and less than 12 weeks (group D).

XELOX (capecitabine: 1000 mg/m^2^, per os twice daily, days 1–14; oxaliplatin: 130 mg/m^2^, intravenous infusion over 2 h in 250 mL dextrose 5%, day 1. Administered every 3 weeks). SOX (S-1: 40–60 mg [BSA < 1.25 m^2^, 40 mg; 1.25 m^2^ ≤ BSA ≤ 1.5 m^2^, 50 mg; BSA > 1.5 m^2^, 60 mg], per os twice daily, days 1–14; oxaliplatin: 130 mg/m^2^, intravenous infusion over 2 h in 250 mL dextrose 5%, day 1. Administered every 3 weeks). FOLFOX (oxaliplatin: 85 mg/m^2^, intravenous infusion over 2 h in 250 mL dextrose 5%, day 1; leucovorin calcium, 400 mg/m^2^, intravenous infusion over 2 h in 250 mL dextrose 5%, followed by FU, 400 mg/m^2^ administered as a bolus injection [intravenous push administered by hand] and then 2400 mg/m^2^ administered as an intravenous infusion over 46 h. Administered every 2 weeks). Adverse events were assessed according to the National Cancer Institute’s Common Terminology Criteria for Adverse Events (version 3.0).

OS, 5-year OS rate were (co) primary endpoints. DFS, 5-year DFS rate were secondary endpoints. All statistical analyses were performed using SPSS software 22.0 (SPSS Inc., Chicago, IL, USA). Chi-squared tests (Pearson’s chi-squared, linear-by-linear association) and one-way ANOVA (post hoc multiple comparisons) were performed to compare the continuous and categorical variables among the four groups. Univariate and multivariate analyses were performed using the Cox proportional hazards model. The prognostic factors included in the multivariate survival analysis using the forward stepwise method were age, surgical approach, intraoperative blood loss, postoperative complications, tumor length/diameter, histological type, vascular tumor embolus, depth of invasion, lymph node metastasis, TNM stage, serum CA19–9 level, and number of adjuvant chemotherapy cycles. A two-sided *P* < 0.05 was considered statistically significant. Survival analysis (OS and DFS) was performed using the Kaplan–Meier method, and the log-rank test for pairwise comparisons over strata was performed for the four groups. According to the Bonferroni method, α was adjusted by dividing 0.05 by 6, and two-sided *P* < 0.008 was considered statistically significant.

## Results

**Fig. 1:**
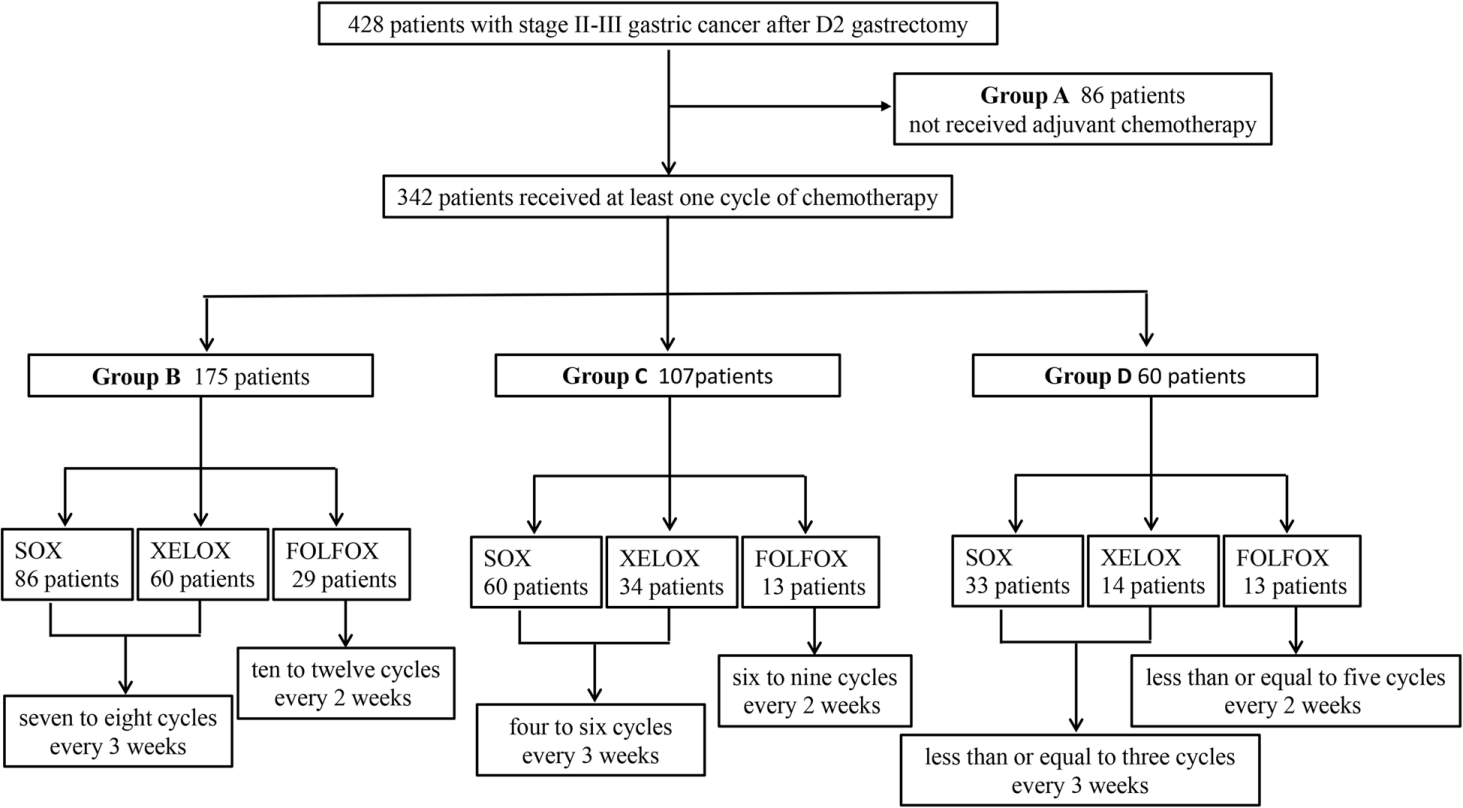
Duration and regimens of adjuvant chemotherapy for II-III GC patients

In total, 428 patients with stage II–III GC who underwent D2 gastrectomy were finally analyzed. Of these, 86 patients did not receive adjuvant chemotherapy (group A), and 342 patients received at least one cycle doublet chemotherapy. One hundred seventy-five patients completed 7–8 cycles of adjuvant chemotherapy administered every 3 weeks or 10–12 cycles of chemotherapy administered every 2 weeks (group B). Additionally, 107 patients completed 4–6 cycles of chemotherapy administered every 3 weeks or 6–9 cycles of chemotherapy administered every 2 weeks (group C), and 60 patients received up to 3 cycles of chemotherapy administered every 3 weeks or up to 5 cycles of chemotherapy administered every 2 weeks (group D). The specific chemotherapy regimens and durations are shown in Fig. 1. The clinicopathological characteristics of the four groups are listed in Table 1. The data were well balanced among the groups excluding age (*P* < 0.001), BMI (*P* = 0.046), postoperative complications (*P* = 0.001) and tumor length/diameter (*P* = 0.010). In terms of age, pairwise comparisons among the four groups revealed significant differences, which suggests that patients’ probability to receive chemotherapy and the duration of adjuvant chemotherapy decreased with increasing age. In terms of BMI, a significant difference was only found between groups A and B (median: 22.7 versus 23.9, *P* = 0.008), which suggests that patients with low BMI might be less willing to receive adjuvant chemotherapy than those with high BMI. In terms of postoperative complications, pairwise comparisons revealed significant differences between group A and the other three groups, whereas no differences were observed among the other three groups. This suggests that the existence of postoperative complications affected patients’ probability to receive adjuvant chemotherapy, but not the duration of treatment. Concerning tumor length/diameter, we found no association with adjuvant chemotherapy (Table 2).

**Table 1:** Baseline characteristics

Variables	Total	Adjuvant chemotherapy cycles					
		Not received (A)	Received (cycles)				*P*
			7–8 or 10–12 cycles (B)	4–6 or 6–9 cycles (C)	≤3 or 5 cycles (D)	Total	
n	428	86 (20.1)	175 (40.9)	107 (25.0)	60 (14.0)	342 (79.9)	
Age (years)							< 0.001
Median	60	66	57	60	62	59	
Range	23–79	38–79	25–78	23–78	41–79	23–79	
Sex							0.458
Female	124 (29.0)	26 (30.2)	49 (28.0)	27 (25.2)	22 (36.7)	98 (28.7)	
Male	304 (71.0)	60 (69.8)	126 (72.0)	80 (74.8)	38 (63.3)	244 (71.3)	
BMI							0.046
Median	23.4	22.7	23.9	22.6	23.8	23.5	
Range	14.8–32.0	15.9–31.2	16.5–32.0	14.8–31.1	17.1–29.4	14.8–32.0	
Surgical approach							0.409
Traditional open	128 (29.9)	22 (25.6)	49 (28.0)	37 (34.6)	20 (33.3)	106 (31.0)	
Laparoscopic-assisted	286 (66.8)	62 (72.1)	121 (69.1)	67 (62.6)	36 (60.0)	224 (65.5)	
Conversion to open	14 (3.3)	2 (2.3)	5 (2.9)	3 (2.8)	4 (6.7)	12 (3.5)	
Intraoperative blood loss							0.323
≤ 100 ml	351 (82.0)	71 (82.6)	137 (78.3)	93 (86.9)	50 (83.3)	280 (81.9)	
>100 ml	77 (18.0)	15 (17.4)	38 (21.7)	14 (13.1)	10 (16.7)	62 (18.1)	
Postoperative complications							0.001
Negative	376 (87.9)	65 (75.6)	162 (92.6)	96 (89.7)	53 (88.3)	311 (90.9)	
Positive	52 (12.1)	21 (24.4)	13 (7.4)	11 (10.3)	7 (11.7)	31 (9.1)	
Tumor length/diameter (cm)							0.010
Median	4.2	4.2	4.0	4.0	5.0	4.2	
Range	0.3–19.0	1.5–14.3	0.3–19.0	1.5–14.0	2.0–13.0	0.3–19.0	
Histological type							0.511
High differentiation adenocarcinoma	3 (0.7)	1 (1.2)	1 (0.6)	0 (0.0)	1 (1.7)	2 (0.6)	
Median differentiation adenocarcinoma	84 (19.6)	22 (25.6)	31 (17.7)	21 (19.6)	10 (16.7)	62 (18.1)	
Low differentiation adenocarcinoma	173 (40.4)	26 (30.2)	78 (44.6)	44 (41.1)	25 (41.7)	147 (43.0)	
Median-Low differentiation adenocarcinoma	132(30.8)	30 (34.9)	47 (26.9)	35 (32.7)	20 (33.3)	102 (29.8)	
Mucinous adenocarcinoma	14 (3.3)	4 (4.7)	5 (2.9)	3 (2.8)	2 (3.3)	10 (2.9)	
Signet-ring cell carcinoma	22 (5.1)	3 (3.5)	13 (7.4)	4 (3.7)	2 (3.3)	19 (5.6)	
Vascular tumor embolus							0.080
Negative	183 (42.8)	46 (53.5)	75 (42.9)	42 (39.3)	20 (33.3)	137 (40.1)	
Positive	245 (57.2)	40 (46.5)	100 (57.1)	65 (60.7)	40 (66.7)	205 (59.9)	
Depth of Invasion (T)							0.661
T1	4 (0.9)	0 (0.0)	2 (1.1)	1 (0.9)	1 (1.7)	4 (0.9)	
T2	46 (10.7)	6 (7.0)	19 (10.9)	15 (14.0)	6 (10.0)	40 (11.7)	
T3	177 (41.4)	40 (46.5)	74 (42.3)	41 (38.3)	22 (36.7)	137 (40.1)	
T4a	194 (45.3)	38 (44.2)	78 (44.6)	49 (45.8)	29 (48.3)	156 (45.6)	
T4b	7 (1.6)	2 (2.3)	2 (1.1)	1 (0.9)	2 (3.3)	5 (1.5)	
Lymph node metastasis (N)							0.092
N0	91 (21.3)	26 (30.2)	38 (21.7)	18 (16.8)	9 (15.0)	65 (19.0)	
N1	108 (25.2)	17 (19.8)	43 (24.6)	33 (30.8)	15 (25.0)	91 (26.6)	
N2	105 (24.5)	26 (30.2)	38 (21.7)	28 (26.2)	13 (21.7)	79 (23.1)	
N3a	90 (21.0)	11 (12.8)	39 (22.3)	23 (21.5)	17 (28.3)	79 (23.1)	
N3b	34 (7.9)	6 (7.0)	17 (9.7)	5 (4.7)	6 (10.0)	28 (8.2)	
TNM							0.585
II	166 (38.8)	37 (43.0)	68 (38.9)	42 (39.3)	19 (31.7)	129 (37.7)	
IIA	91 (21.3)	20 (23.3)	38 (21.7)	23 (21.5)	10 (16.7)		
IIB	75 (17.5)	17 (19.8)	30 (17.1)	19 (17.8)	9 (15.0)		
III	262 (61.2)	49 (57.0)	107 (61.1)	65 (60.7)	41 (68.3)	213 (62.3)	
IIIA	139 (32.5)	30 (34.9)	54 (30.9)	36 (33.6)	19 (31.7)		
IIIB	89 (20.8)	13 (15.1)	36 (20.6)	24 (22.4)	16 (26.7)		
IIIC	34 (7.9)	6 (7.0)	17 (9.7)	5 (4.7)	6 (10.0)		
Serum CEA level							0.184
≤ 5 ng/ml	340 (79.4)	65 (75.6)	148 (84.6)	82 (76.6)	45 (75.0)	275 (80.4)	
>5 ng/ml	88 (20.6)	21 (24.4)	27 (15.4)	25 (23.4)	15 (25.0)	67 (19.6)	
Serum CA199 level							0.272
≤ 37 U/ml	371 (86.7)	71 (82.6)	156 (89.1)	95 (88.8)	49 (81.7)	300 (87.7)	
>37 U/ml	57 (13.3)	15 (17.4)	19 (10.9)	12 (11.2)	11 (18.3)	42 (12.3)	
Recurrence							0.008
Negative	262 (61.2)	43 (50.0)	119 (68.0)	70 (65.4)	30 (50.0)	219 (64.0)	
Positive	166 (38.8)	43 (50.0)	56 (32.0)	37 (34.6)	30 (50.0)	123 (36.0)	
Survival							< 0.001
Alive	283 (66.1)	45 (52.3)	129 (73.7)	77 (72.0)	32 (53.3)	238 (69.6)	
Dead	145 (33.9)	41 (47.7)	46 (26.3)	30 (28.0)	28 (46.7)	104 (30.4)	

Note: *P* < 0.05, statistically significant; *P* values between groups A, B, C and D

**Table 2:** Baseline characteristics

Variables	Adjuvant chemotherapy cycles	Not received (A)	7–8 or 10–12 cycles (B)	4–6 or 6–9 cycles (C)	≤3 or 5 cycles (D)
		*P*	*P*	*P*	*P*
Age	Not received (A)		< 0.001	< 0.001	0.019
	7–8 or 10–12 cycles (B)	< 0.001		0.043	< 0.001
	4–6 or 6–9 cycles (C)	< 0.001	0.043		0.060
	≤3 or 5 cycles (D)	0.019	< 0.001	0.060	
BMI	Not received (A)		0.008	0.356	0.095
	7–8 or 10–12 cycles (B)	0.008		0.080	0.650
	4–6 or 6–9 cycles (C)	0.356	0.080		0.361
	≤3 or 5 cycles (D)	0.095	0.650	0.361	
Postoperative complications	Not received (A)		< 0.001	0.009	0.054
	7–8 or 10–12 cycles (B)	< 0.001		0.405	0.310
	4–6 or 6–9 cycles (C)	0.009	0.405		0.782
	≤3 or 5 cycles (D)	0.054	0.310	0.782	
Tumor length/diameter	Not received (A)		0.361	0.474	0.029
	7–8 or 10–12 cycles (B)	0.361		0.893	0.001
	4–6 or 6–9 cycles (C)	0.474	0.893		0.004
	≤3 or 5 cycles (D)	0.029	0.001	0.004	

Note: *P* < 0.05, statistically significant; P values for pairwise comparisons

**Fig. 2:**
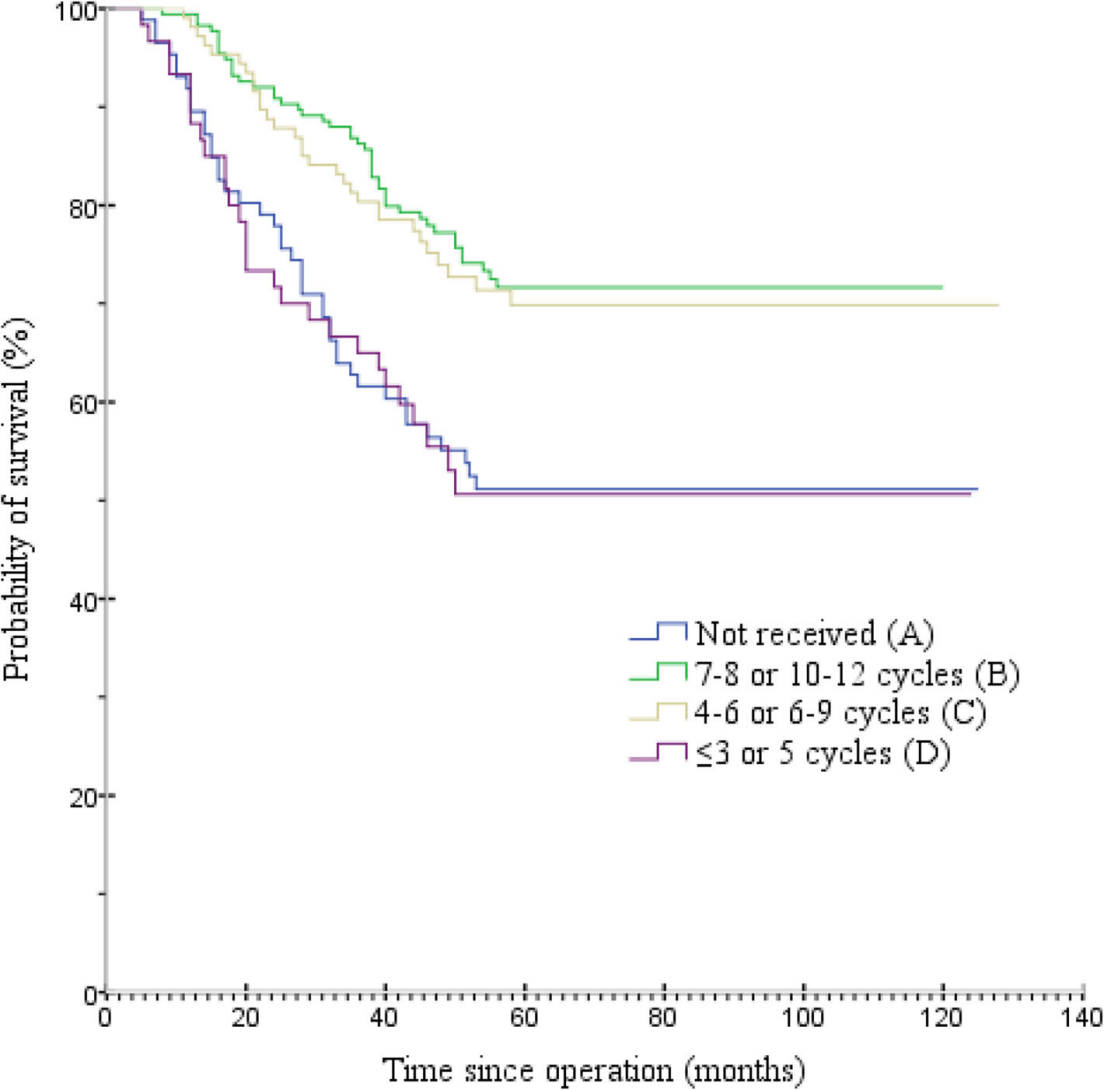
Overall survival by groups A, B, C and D

**Fig. 3:**
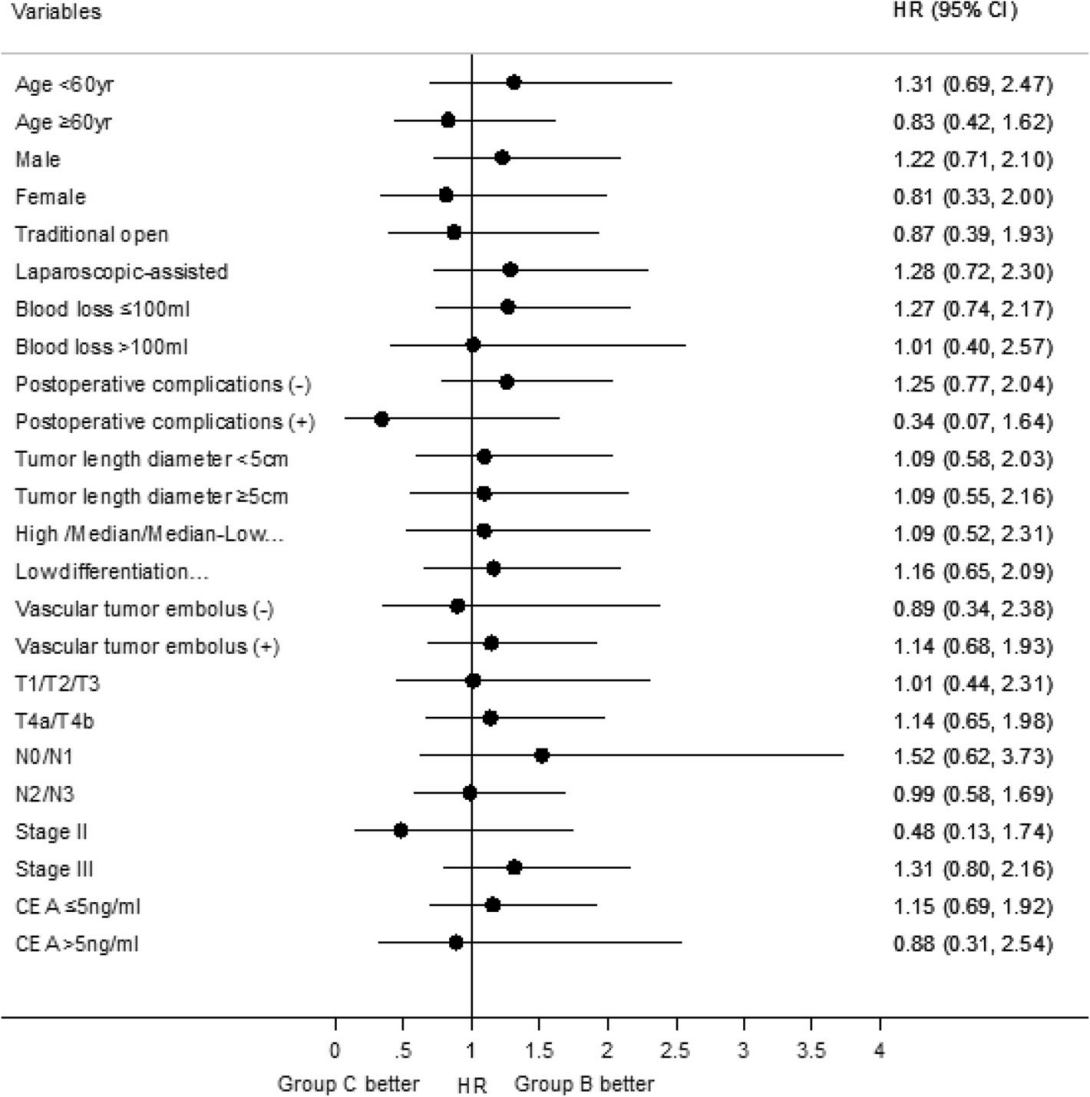
Subgroup analyses on overall survival between groups B and C. High /Median/Median-Low …, High/Median/Median-Low differentiation adenocarcinoma; Low differentiation …, Low differentiation adenocarcinoma/Mucinous adenocarcinoma/Signet-ring cell carcinoma; HR, hazard ratio; CI, confidence interval; *P* < 0.05, statistically significant

**Fig. 4:**
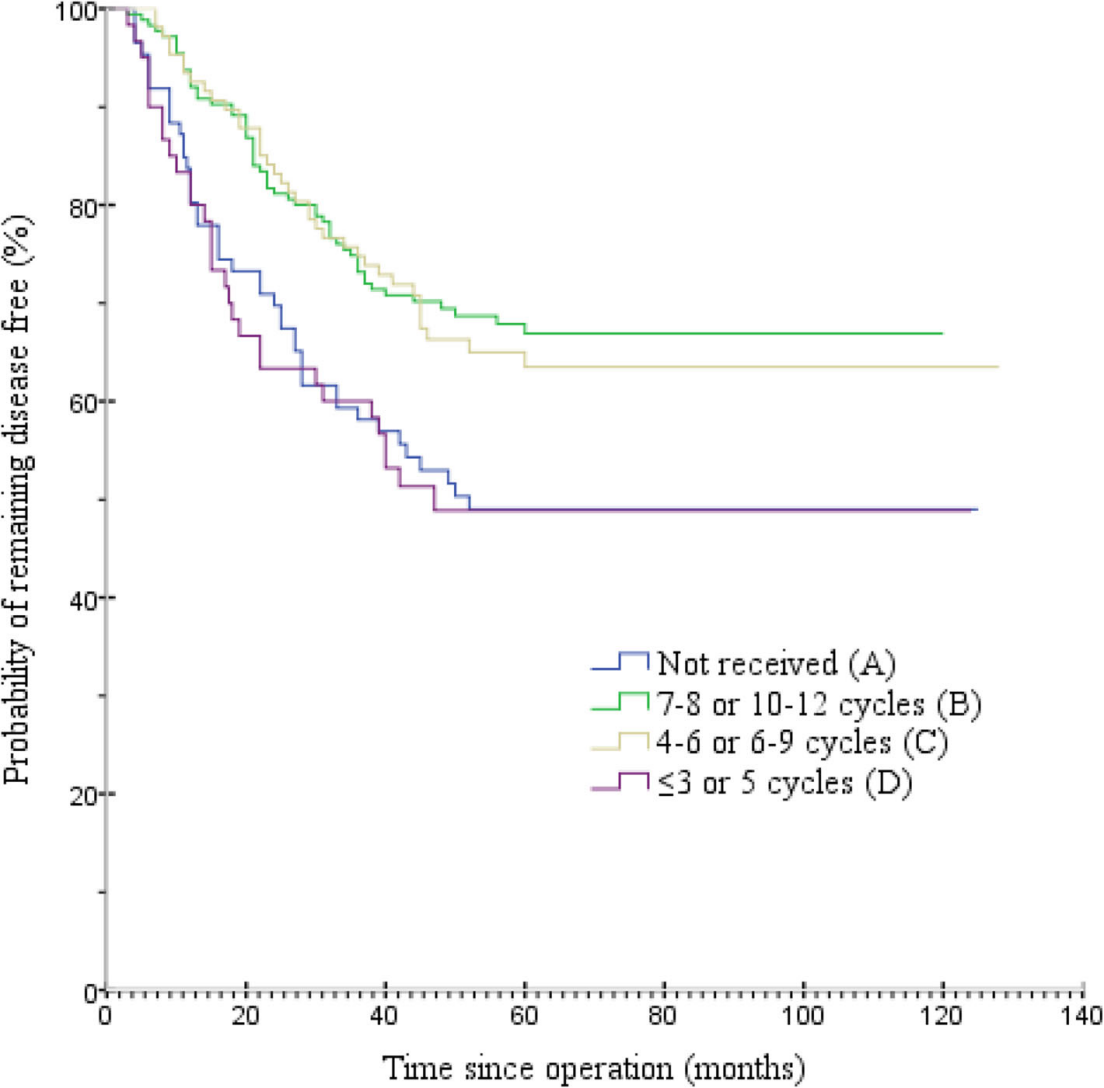
Disease-free survival by groups A, B, C and D

The median follow-up duration was 51 months (range, 5–128 months) in the 428 patients. Figure 2 and Table 3 present OS among the four groups. The median OS for groups A, B, C and D were 47.0, 55.0, 53.0, and 43.0 months, respectively. The 5-year OS rates for groups A, B, C and D were 52.3 (95% confidence interval [CI] = 41.6–63.1%), 73.7 (95% CI = 67.1–80.3%), 72.0 (95% CI = 63.3–80.6%), and 53.3% (95% CI = 40.3–66.3%), respectively. The OS rate was significantly higher in group B than in groups A and D (both *P* < 0.001). Similarly, patients in group C had higher OS rates than in groups A and D (*P* = 0.004 and *P* = 0.005, respectively). There was no significant difference in the OS rate between groups B and C (*P* = 0.677) or between groups A and D (*P* = 0.924). Subgroup analysis was conducted in groups B and C for OS, and no significant differences were found between the two groups (Fig. 3). Figure 4 and Table 4 present the DFS among the four groups. The median DFS for groups A, B, C and D were 41.5, 52.0, 50.0, and 40.5 months, respectively. The 5-year DFS rates for groups A, B, C and D were 50 (95% CI = 39.2–60.8%), 68.0 (95% CI = 61.0–75.0%), 65.4 (95% CI = 56.3–74.6%), and 50.0% (95% CI = 37.0–63.0%), respectively. The DFS rate was significantly higher in group B than in groups A and D (both *P* = 0.003). Similarly, the DFS rate was significantly higher in group C than those in groups A and D (*P* = 0.018 and *P* = 0.015, respectively). Meanwhile, there was no significant difference in DFS rates between groups B and C (*P* = 0.716) or between groups A and D (*P* = 0.838). Based on the aforementioned results, OS (hazard ratio [HR] = 1.277; 95% CI = 0.798–2.042; *P* = 0.308) and DFS (HR = 1.233; 95% CI = 0.811–1.874; *P* = 0.327) were not inferior in group C than those in group B. On multivariate analyses, age, intraoperative blood loss, TNM stage, and the number of adjuvant chemotherapy cycles had independent prognostic significance for OS (Table 5), and intraoperative blood loss, TNM stage, and the number of adjuvant chemotherapy cycles had independent prognostic significance for DFS (Table 6). Tumor length/diameter displayed a trend toward independent prognostic significance for DFS (*P* = 0.053).

**Table 3:** Overall survival by groups A, B, C and D

Adjuvant Chemotherapy cycles	Not received (A)	7–8 or 10–12 cycles (B)	4–6 or 6–9 cycles (C)	≤3 or 5 cycles (D)
	*P*	*P*	*P*	*P*
Not received (A)		< 0.001	0.004	0.924
7–8 or 10–12 cycles (B)	< 0.001		0.677	< 0.001
4–6 or 6–9 cycles (C)	0.004	0.677		0.005
≤3 or 5 cycles (D)	0.924	< 0.001	0.005	

Note: According to the Bonferroni method, α was adjusted by dividing 0.05 by 6, *P* < 0.008, statistically significant

**Table 4:** Disease-free survival by groups A, B, C and D

Adjuvant Chemotherapy cycles	Not received (A)	7–8 or 10–12 cycles (B)	4–6 or 6–9 cycles (C)	≤3 or 5 cycles (D)
	*P*	*P*	*P*	*P*
Not received (A)		0.003	0.018	0.838
7–8 or 10–12 cycles (B)	0.003		0.716	0.003
4–6 or 6–9 cycles (C)	0.018	0.716		0.015
≤3 or 5 cycles (D)	0.838	0.003	0.015	

Note: According to the Bonferroni method, α was adjusted by dividing 0.05 by 6, *P* < 0.008, statistically significant

**Table 5:** Univariate and multivariate analysis of clinicopathologic factors for OS

Variables	Univariate			Multivariate		
	HR	95% CI	*P*	HR	95% CI	*P*
Age (years)						
< 60 yr	1.000			1.000		
≥ 60 yr	1.540	1.107–2.141	0.010	1.418	1.000–2.011	0.050
Sex						
Male	1.000					
Female	1.105	0.777–1.571	0.577			
BMI						
< 23	1.000					
≥ 23	1.020	0.735–1.417	0.905			
Surgical approach						
Traditional open	1.000					
Laparoscopic-assisted	0.918	0.640–1.318	0.644			0.769
Intraoperative blood loss						
≤ 100 ml	1.000			1.000		
>100 ml	1.740	1.191–2.527	0.004	1.780	1.211–2.617	0.003
Postoperative complications						
Negative	1.000					
Positive	1.551	1.001–2.404	0.050			0.729
Tumor length/diameter						
< 5 cm	1.000					
≥ 5 cm	1.738	1.254–2.408	0.001			0.288
Histological type						
HD/MD/M-LD	1.000					
LD/MA/SRC	1.489	1.072–2.068	0.018			0.094
Vascular tumor embolus						
Negative	1.000					
Positive	1.956	1.372–2.789	< 0.001			0.626
Depth of Invasion (T)						
T1/T2/T3	1.000					
T4a/T4b	2.133	1.525–2.983	< 0.001			0.526
Lymph node metastasis (N)						
N0/N1	1.000					
N2/N3	3.247	2.227–4.736	< 0.001			0.336
TNM			< 0.001			< 0.001
IIA	1.000					
IIB	2.905	1.263–6.680	0.012	2.667	1.158–6.142	0.021
IIIA	4.375	2.067–9.259	< 0.001	4.579	2.162–9.697	< 0.001
IIIB	8.092	3.817–17.156	< 0.001	9.092	4.277–19.327	< 0.001
IIIC	14.987	6.776–33.145	< 0.001	16.948	7.627–37.662	< 0.001
Serum CEA level						
≤ 5 ng/ml	1.000					
>5 ng/ml	1.183	0.802–1.744	0.396			
Serum CA199 level						
≤ 37 U/ml	1.000					
>37 U/ml	1.333	1.082–1.644	0.007			0.981
Adjuvant chemotherapy cycles			< 0.001			< 0.001
Not received (A)	1.000			1.000		
7–8 or 10–12 cycles (B)	0.459	0.301–0.699	< 0.001	0.386	0.247–0.604	< 0.001
4–6 or 6–9 cycles (C)	0.504	0.314–0.807	0.004	0.493	0.304–0.800	0.004
≤ 3 or 5 cycles (D)	1.032	0.638–1.670	0.897	0.870	0.530–1.427	0.580

Note: *HD/MD/M-LD* High/Median/Median-Low differentiation adenocarcinoma, *LD/MA/SRC* Low differentiation adenocarcinoma/Mucinous adeno-carcinoma/ Signet-ring cell carcinoma, *HR* Hazard ratio, *CI* Confidence interval. *P* < 0.05, statistically significant

**Table 6:** Univariate and multivariate analysis of clinicopathologic factors for DFS

Variables	Univariate			Multivariate		
	HR	95% CI	*P*	HR	95% CI	*P*
Age (years)						
< 60 yr	1.000					
≥ 60 yr	1.297	0.955–1.760	0.096			0.301
Sex						
Male	1.000					
Female	1.208	0.873–1.671	0.254			
BMI						
< 23	1.000					
≥ 23	0.970	0.714–1.318	0.847			
Surgical approach						
Traditional open	1.000					
Laparoscopic-assisted	0.802	0.576–1.116	0.191			0.264
Intraoperative blood loss						
≤ 100 ml	1.000			1.000		
>100 ml	1.777	1.252–2.522	0.001	1.829	1.279–2.615	0.001
Postoperative complications						
Negative	1.000					
Positive	1.365	0.892–2.090	0.151			0.838
Tumor length/diameter						
< 5 cm	1.000					
≥ 5 cm	1.929	1.421–2.619	< 0.001			0.053
Histological type						
HD/MD/M-LD	1.000					
LD/MA/SRC	1.506	1.108–2.047	0.009			0.129
Vascular tumor embolus						
Negative	1.000					
Positive	1.779	1.285–2.464	0.001			0.901
Depth of Invasion (T)						
T1/T2/T3	1.000					
T4a/T4b	2.149	1.572–2.939	< 0.001			0.207
Lymph node metastasis (N)						
N0/N1	1.000					
N2/N3	2.650	1.897–3.701	< 0.001			0.690
TNM			< 0.001			< 0.001
IIA						
IIB	2.748	1.380–5.469	0.004	2.634	1.323–5.246	0.006
IIIA	3.216	1.715–6.033	< 0.001	3.347	1.783–6.281	< 0.001
IIIB	6.268	3.340–11.762	< 0.001	6.941	3.687–13.065	< 0.001
IIIC	12.823	6.476–25.392	< 0.001	13.420	6.751–26.677	< 0.001
Serum CEA level						
≤ 5 ng/ml	1.000					
>5 ng/ml	1.022	0.701–1.490	0.911			
Serum CA199 level						
≤ 37 U/ml	1.000					
>37 U/ml	1.291	1.057–1.578	0.012			0.840
Adjuvant chemotherapy cycles			0.002			< 0.001
Not received (A)	1.000			1.000		
7–8 or 10–12 cycles (B)	0.549	0.369–0.817	0.003	0.424	0.283–0.635	< 0.001
4–6 or 6–9 cycles (C)	0.592	0.381–0.918	0.019	0.522	0.335–0.815	0.004
≤ 3 or 5 cycles (D)	1.058	0.664–1.687	0.812	0.836	0.519–1.349	0.464

Note: *HD/MD/M-LD* High/Median/Median-Low differentiation adenocarcinoma, *LD/MA/SRC* Low differentiation adenocarcinoma/Mucinous adeno-carcinoma/ Signet-ring cell carcinoma, *HR* Hazard ratio, *CI* Confidence interval. *P* < 0.05, statistically significant

Of the 342 patients who received chemotherapy, no treatment-related death occurred, and 21 were excluded from the safety population due to the absence of toxicity-related follow-up information (10 in group B, 8 in group C, and 3 in group D). The most common grade 3 or 4 adverse events were neutropenia, thrombocytopenia and nausea/vomiting (Table 7), and there were no significant differences among the three groups. Grade 3 or 4 neutropenia occurred in 33 (20.0%) patients in group B, 19 (19.2%) patients in group C, and 8 (14.0%) patients in group D.

**Table 7:** Adverse events observed among groups B, C and D

Adverse event	Grade 3 or 4, n (%)				*p*
	group B (*n* = 165)	group C (*n* = 99)	group D (*n* = 57)	total (*n* = 321)	
Nausea/Vomiting	26 (15.8)	16 (16.2)	10 (17.5)	52 (16.2)	0.951
Neutropenia	33 (20.0)	19 (19.2)	8 (14.0)	60 (18.7)	0.602
Thrombocytopenia	10 (6.1)	8 (8.1)	3 (5.3)	21 (6.5)	0.985

## Discussion

The MAGIC, ACTS-GC, and CLASSIC trials have provided high-level evidence of the benefit of adjuvant chemotherapy in GC [5–7]. However, there is no global agreement on chemotherapy regimens and durations for GC. Although 6 months of XELOX, SOX, FOLFOX treatment or eight 6-week cycles of S-1 are recommended for patients with advanced GC after D2 gastrectomy in China [8], many patients cannot complete the full course of treatment because of adverse events. In the ACTS-GC study, only 340 (65.8%) of the 517 patients receiving S-1 continued treatment for 12 months, including 158 (46.5%) dose reductions [15]. In the CLASSIC trial, only 346 patients (67%) competed eight cycles as planned. Moreover, 48% of patients required capecitabine dose reductions, and 47% required oxaliplatin dose reductions. Ninety percent of patients required dose modification because of adverse events, and the most common adverse events in the chemotherapy group were nausea, vomiting, neutropenia, decreased appetite, diarrhea, and peripheral neuropathy. Peripheral neuropathy, a cumulative dose-limiting toxicity associated with oxaliplatin, occurred in 56% of patients who received chemotherapy [7]. Neurotoxicity usually peaks within a few months after the last dose of oxaliplatin, making it difficult to personalize treatment with an empirical dose. Such toxic effects can last long after treatment and severely affect daily living activities such as writing, dressing and handling objects [16]. Given the cumulative, dose-dependent oxaliplatin-induced neurotoxicity, patients may benefit from a shorter duration of adjuvant treatment without sacrificing efficacy. In our study, for patients with stage II–III GC after D2 gastrectomy, the 5-year OS rates for groups A, B, C and D were 52.3, 73.7, 72.0, and 53.3%, respectively. The results suggest that shortening the duration of adjuvant chemotherapy to 4–6 cycles administered every 3 weeks or 6–9 cycles administered every 2 weeks (group C) produced similar efficacy as 7–8 cycles administered every 3 weeks or 10–12 cycles administered every 2 weeks (group B). No treatment (group A) or early termination of postoperative treatment (group D) was associated with worse OS. Subgroup analysis found no statistical difference between groups B and C concerning OS. The multivariate analysis confirmed with high statistical significance the efficacy of complete courses of adjuvant chemotherapy, and, among them, the similar impact of 4–6/6–9 and 7–8/10–12 cycles, resulting in similar HRs vs Group A (0.52 and 0.42, respectively). However, we should note that the observed HRs for OS and DFS between groups B (*n* = 175) and C (*n* = 107) are quite consistent (1.27 and 1.23, respectively), favoring group B, and quite far from 1.00. Indeed, in Figs. 2 and 4 the OS and DFS curves are very near yet not totally overlapping. The observed lack of statistical significance may be due to the small sample size of the groups, particularly when subgroups analysis.

The addition of eight cycles of oral capecitabine to the eight-cycle XELOX regimen did not significantly improve 3-year OS in patients with stage II–III gastric cancer [9]. Similarly, prolonged postoperative chemotherapy for less than 1 year, less than 2 years, or more than 2 years did not significantly improve survival [12]. However, more patients in the prolonged group experienced more adverse events [9, 12]. Three months of treatment with CAPOX has been introduced for patients with stage III colon cancer in the lower-risk group based on the IDEA collaboration, a large-scale, prospective, pooled analysis of phase 3 trials. As expected, a shorter duration of treatment significantly reduced the incidence and severity of adverse events, without sacrificing efficacy [14]. Qu et al. [13] conducted a retrospective analysis of 237 patients with stage IB–IIIC GC who received four, six, or eight cycles of FU plus oxaliplatin, FU plus non-oxaliplatin combinations, or FU monotherapy after D1 or D2 radical gastrectomy. The 5-year OS rates for eight, six, and four cycles were 65.8, 74.0, and 41.2%, respectively, which illustrates that six cycles of FU-based adjuvant chemotherapy are adequate. We retrospectively analyzed 428 patients with stage II–III GC after D2 gastrectomy, and the 5-year OS rates for groups B and C were 73.7 and 72.0%, respectively, which were higher than those of patients who completed eight cycles and comparable to those of patients who completed six cycles in the study by Qu et al.. Our results were also comparable with those of the ACTS-GC study, in which all patients with confirmed stage II–III gastric cancer underwent D2 gastrectomy, and the 5-year OS rate was 71.7% in the S-1 group [6]. To reduce toxicity while maintaining efficacy, patients should avoid the two additional cycles of FU plus oxaliplatin without worrying about adverse outcomes. Regarding S-1 monotherapy, JCOG1104 [OPAS-1], an open-label, phase 3, non-inferiority, randomized trial, found that four courses of S-1 (treatment lasted for 6 months) was inferior to eight courses of S-1 (treatment lasted for 1 year) concerning relapse-free survival (RFS) among patients with confirmed stage II GC [11]. Hence, eight courses of S-1 remain the standard treatment for stage II GC.

It is important to note that all patients in our study underwent D2 gastrectomy, and thus, the optimal treatment after D0 or D1 gastrectomy may be different. The INT-0116 study, in which 36% of patients underwent D1 lymph node dissection and 54% underwent D0 lymph node dissection, found that postoperative chemoradiotherapy significantly improved RFS and OS in patients with GC. The updated analysis with a median of more than 10 years of follow-up revealed a strong persistent benefit [4, 17]. However, the intergroup CALGB 80101 trial demonstrated that more intensive systemic chemotherapy combined with postoperative chemoradiotherapy (as shown in INT-0116) produced no survival benefit [18]. Moreover, the ARTIST trial failed to demonstrate that the addition of radiotherapy to postoperative adjuvant chemotherapy significantly improved DFS in patients who underwent D2 gastrectomy [19]. Therefore, it appears that postoperative chemoradiotherapy can compensate for inadequate surgery.

No survival difference was found between traditional open and laparoscopic-assisted approaches in our study. Regarding short-term outcomes, laparoscopic-assisted D2 gastrectomy proved feasible compared with traditional open surgery in some randomized controlled trials, but the long-term efficacy has not been clarified [20, 21]. Our study found that patients older than 60 years were less likely to survive than those younger than 60 years, suggesting that age affects the survival of GC [22, 23]. To our surprise, blood loss exceeding 100 mL during surgery was a risk factor for both overall mortality and recurrence in univariate and multivariate analyses. This finding is consistent with many research results that operative blood loss predicts worse survival in patients undergoing surgery [24–26], which has also been reported for a variety of other malignancies, such as pancreatic [27, 28], colorectal [29–31], and lung cancers [32]. BMI, tumor size, year of surgery, and excision extension were associated with increased blood loss [24, 25, 29]. Our research did not analyze data concerning the time between surgery and adjuvant chemotherapy, which did not exceed 3 months as per inclusion criteria. From previous studies, the duration of adjuvant chemotherapy, but not the period between surgery and chemotherapy, affected OS in GC [33, 34].

Some limitations of this study should be considered. First, as a retrospective study, our ability to obtain detailed data regarding short- and long-term chemotherapy-related adverse events and dose reduction was limited, especially concerning peripheral neuropathy. In the CLASSIC trial, the incidence of all adverse events was as high as 99% among 496 patients in the chemotherapy group. Although different grade 3 or 4 adverse events were infrequent, ranging in incidence from less than 1 to 22%, the cumulative incidence was also as high as 56% in 496 patients [7]. Because some adverse events are associated with survival [35, 36], further studies are needed to confirm the impact of those adverse events on intergroup survival differences. Second, patients in our research received one of three different chemotherapy regimens (SOX, XELOX, and FOLFOX). However, there is a lack of prospective studies to determine which regimen is superior. Current studies suggest that XELOX regimen does not result in a greater survival benefit compared with FOLFOX6 regimen [37], and the SOX therapy has similar survival benefits to XELOX in Chinese patients with GC following D2 gastrectomy [38]. Additionally, since groups B, C, and D were balanced concerning the receipt of these three regimens, it is likely that differences in the chemotherapy regimen do not explain the differences in survival among the groups. Third, although all patients in the study were collected consecutively based on inclusion and exclusion criteria, there was indeed a lack of fair matching of clinical parameters (Age, BMI, Postoperative complications and Tumor length/diameter) between groups, mainly between group A and the other three groups. For patients in group A, 44 patients refused chemotherapy due to personal willingness, 21 patients did not receive chemotherapy due to advanced age (65 years or older), and 10 patients due to self-conscious physical weakness, and 11 patients refused chemotherapy after recovery from complications (gastroparesis or anastomotic fistula). However, all these patients recovered to Eastern Cooperative Oncology Group (ECOG) score of 0–1 within 3 months after the operation. This cohort study suggested that age ≥ 60 years was an independent risk factor for OS, which may have had significant intergroup influence on prognosis, while BMI, tumor length/diameter and postoperative complications were not independent prognostic factors. Notably, median age was ≥60 years in groups A and D, and < 60 years in groups B and C. While group C displayed higher median age than group B, survival was similar, which confirmed the feasibility of shortened adjuvant chemotherapy duration from another aspect. Likewise, group D displayed lower median age than group A, but survival was similar.

To our knowledge, we conducted the first analysis on duration of adjuvant chemotherapy in this setting. Given the limited numerosity, the monocentric nature, and the limitations inherent to retrospective collection, including treatment heterogeneity and chance of selection bias, its results should be interpreted as preliminary and hypothesis-generating. Hopefully, further studies, larger in size, and prospective and randomized in design, will fully elucidate the impact of treatment duration in this setting. If confirmed, the non-inferiority of shorter treatment courses could spare patients costs and unnecessary toxicities.

## Conclusions

In conclusion, to reduce toxicity without decreasing efficacy, 4–6 cycles of XELOX or SOX chemotherapy regimens administered every 3 weeks or 6–9 cycles of FOLFOX administered every 2 weeks (group C) might be a favorable option for patients with stage II–III GC after radical gastrectomy with D2 lymphadenectomy. Prospective multicenter clinical trials with adequate sample sizes are necessary. Based on the IDEA trial, a multi-center, randomized, parallel assignment clinical trial named LOMAC is underway in China with a target enrollment of 1032 participants with stage II, IIIA, or IIIB GC after D2 gastrectomy to verify the hypothesis that CAPOX for 4 months is non-inferior to CAPOX for 6 months concerning DFS and safety. We expect more studies in the future to provide high-level evidence of the optimal duration of adjuvant chemotherapy in GC.

## Data Availability

The datasets used and/or analysed during the current study are available from the corresponding author on reasonable request.

## Abbreviations

GC: Gastric cancer
FU: Fluorouracil
XELOX: Capecitabine and oxaliplatin
SOX: S-1 and oxaliplatin
FOLFOX: Fluorouracil, oxaliplatin and leucovorin calcium
OS: Overall survival
DFS: Disease-free survival
BSA: Body Surface Area
BMI: Body Mass Index
CI: Confidence interval
HR: Hazard ratio
CAPOX: Capecitabine plus oxaliplatin
RFS: Relapse-free survival
ECOG: Eastern Cooperative Oncology Group
